# A Case Report of an Unstable C-spine Fracture in the Emergency Department

**DOI:** 10.21980/J8SK90

**Published:** 2025-04-30

**Authors:** Jinho Jung, Tyler Rigdon, Alisa Wray, Danielle Matonis

**Affiliations:** * University of California, Irvine, School of Medicine, Irvine, CA; ^ University of California, Irvine Medical Center, Department of Emergency Medicine, Orange, CA

## Abstract

Unstable cervical spine (c-spine) fractures are of high concern in traumatic incidents because they may result in significant morbidity and mortality. This is a case of a 44-year-old male who presents to the Emergency Department (ED) with neck pain after recreational wrestling and was found to have an unstable C-spine fracture. His treatment course was complicated by multiple interrupted hospital stays due to leaving against medical advice (AMA) and subsequent returns to the emergency department. The patient received both CT and MRI imaging and ultimately underwent occiput to C3 fusion with drain placement with a favorable outcome. This case report highlights the diagnosis and treatment of a patient with an unstable c-spine fracture. Key lessons from the case include the importance of timely recognition of patients with a potential c-spine fracture and identifying those who are at risk for nonadherence to medical treatment plans in order to provide interventions and improve chances of adherence. For patients in which pre-hospital care is involved, such as emergency medical services (EMS), recognition and appropriate care, such as c-spine stabilization, may be important for long-term outcomes.

**Topics:**

Unstable c-spine fracture, polysubstance use, spinal injury, neck trauma.

**Figure:**
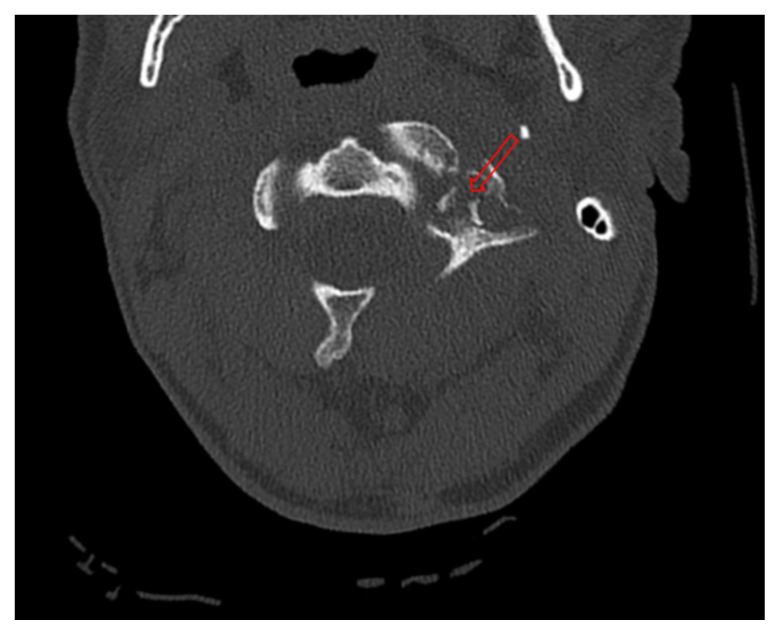
Axial CT Video Link: https://youtu.be/hOogTxYqxto

**Figure:**
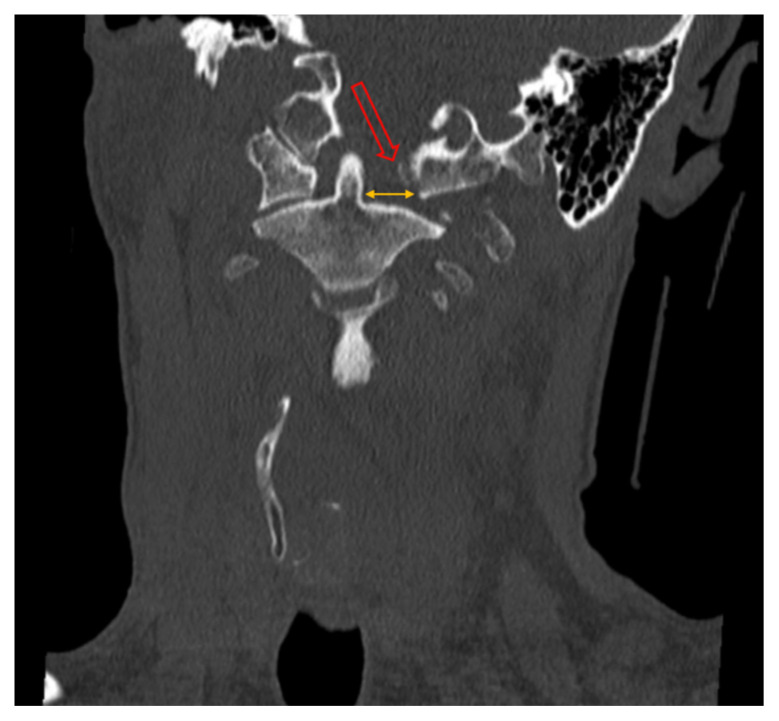
Coronal CT Video Link: https://youtu.be/f3C6Zor7Wes

**Figure:**
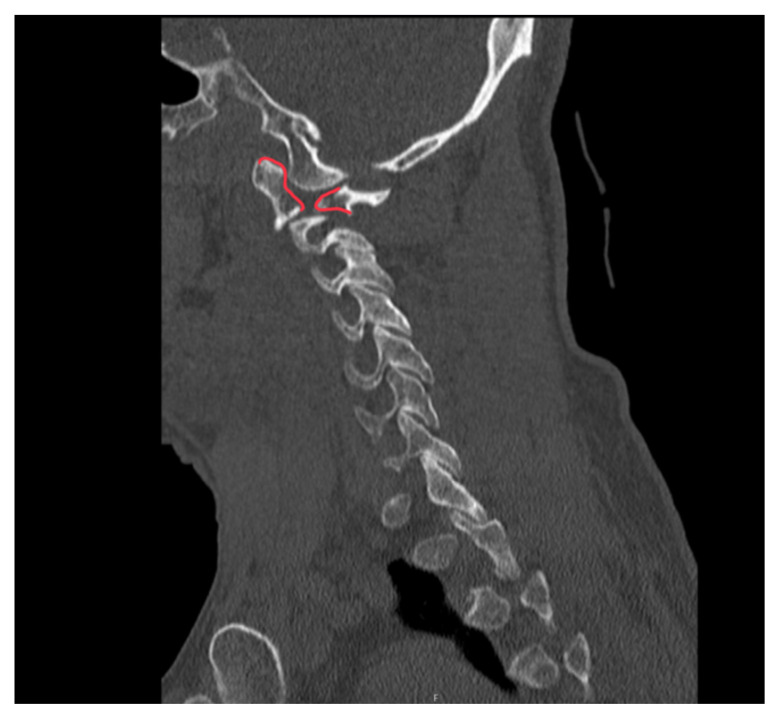
Sagittal CT Video Link: https://youtu.be/m-PII5fD29c

**Figure:**
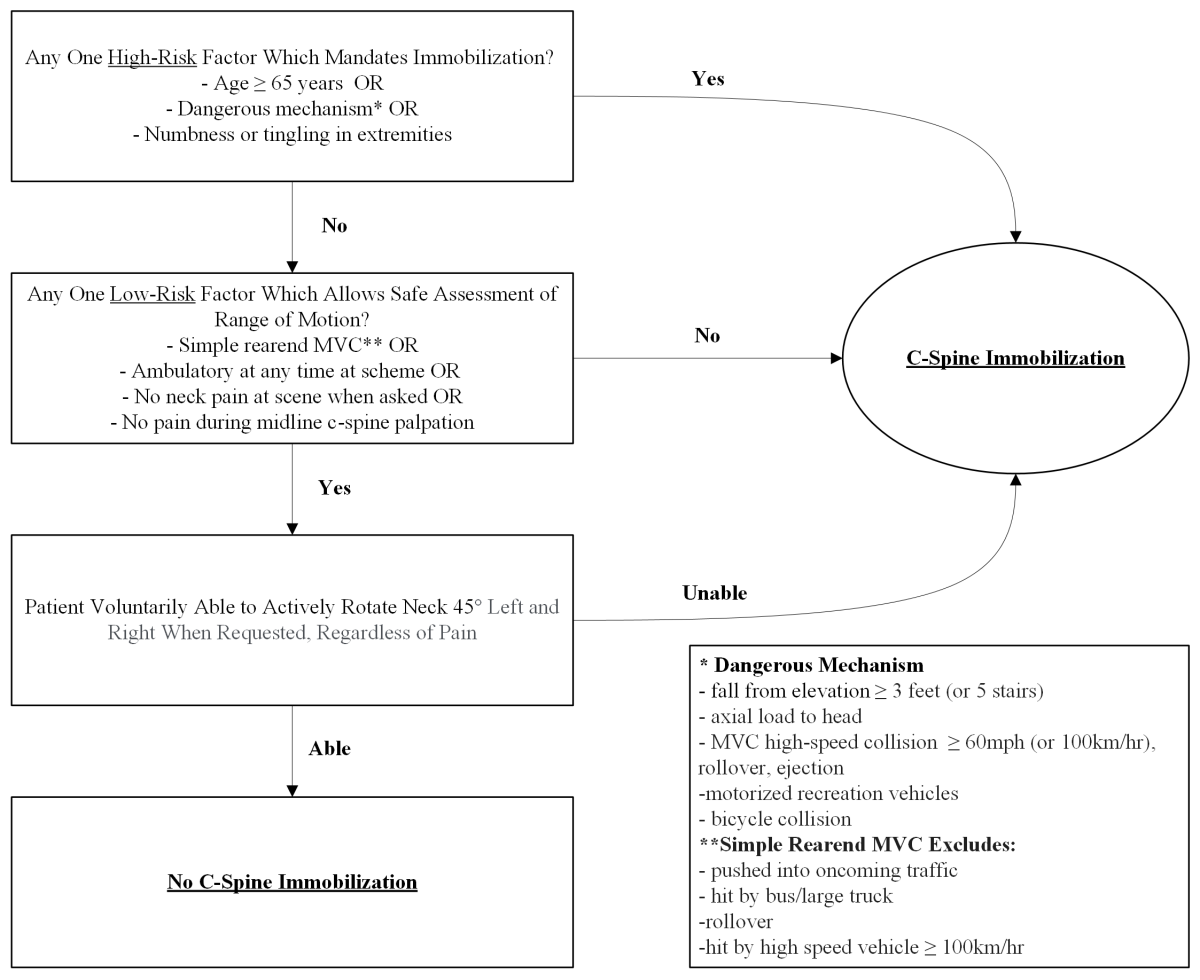


## Brief introduction

Unstable cervical spine (c-spine) fractures are important to identify in situations of potential trauma in the emergency department (ED), particularly in populations at risk for noncompliance with medical care. The prevalence of cervical trauma among spinal trauma patients ranges from 33.7% to 53.7%, with higher rates observed in males, particularly those aged 15 to 30 years.^1^ It is crucial for ED physicians to manage potential cervical spine injuries promptly and effectively to prevent further neurological damage.^2^ We describe a case in which a 44-year-old male with a past medical history of polysubstance use who presents to the ED with an unstable C-spine fracture. His treatment and management were complicated by disjointed care requiring readmission due to the patient’s leaving against medical advice (AMA) as well as polysubstance withdrawal requiring a period of ICU level of care. The patient ultimately underwent surgical fusion of the occiput to C3 with drain placement and had a favorable outcome.

## Presenting concerns and clinical findings

A 44-year-old male with a past medical history of polysubstance use presented to the ED by private transport with a chief complaint of neck pain. The patient reported wrestling with his cousin two days prior when he sustained an injury to his neck after being flipped over onto the ground, hitting his head. He initially presented to a different hospital by EMS but left AMA. The patient reported the previous hospital diagnosed him with a spinal fracture, but he was uncertain of any further details. He did not bring any records or documentation and did not provide a clear reason for leaving the previous ED. His last fentanyl use was six hours prior to arrival. He did endorse periodic IV drug abuse. He denied changes in sensation, weakness, headache, vision changes, ear pain, hearing loss, urinary retention, dysuria, fever/chills, chest pain, shortness of breath, and abdominal pain.

The patient’s initial vitals were remarkable for mild tachycardia and hypertension; however, he was afebrile with normal oxygen saturation on room air. The patient was alert and fully oriented with midline c-spine tenderness. Cranial nerves II through XII were all intact. He had 5/5 strength and intact sensation in all four extremities. No saddle anesthesia was noted. He had a negative Romberg’s test and examination of finger-to-nose and heel-knee-shin was intact. His gait was normal.

## Significant findings

The initial workup in the ED showed an acute displaced fracture of the left occipital condyle (CT-coronal, fracture of the left occipital condyle, red arrow; displacement, orange line), a shattered left lateral mass with involvement of the vertebral canal (CT-axial, red arrow), and malalignment of the craniocervical junction (CT-sagittal, red outline). The CT angiogram head and neck showed a possible irregularity in the left vertebral artery. The CT head without contrast had no significant findings.

## Patient course

The spine surgery team was consulted and agreed on inpatient admission and planned for urgent surgical repair. The patient was admitted to the hospital and placed on Clinical Institute of Withdrawal and Assessment (CIWA) and Clinical Opiate Withdrawal Symptoms (COWS) with social work and pain management consultation. An MRI acute cord survey with contrast redemonstrated the C1 fracture as well as disruption of the craniocervical junction, tear of the anterior and posterior longitudinal ligaments, and edema and fracture of the bilateral occipital condyles. There was no cord compression. The patient was scheduled to go to surgery; however, he chose to leave AMA.

The patient subsequently returned to the ED 4 days later for the same complaint of neck pain and was admitted. During this admission, he was upgraded to ICU level care due to polysubstance withdrawal. He then underwent occiput to C3 fusion with surgical drain placement. The drains were removed 5 days after surgery. The patient was ultimately discharged with hydroxyzine for anxiety, naltrexone for alcohol use disorder, buprenorphine/naloxone for opioid dependence, and a follow-up appointment with a substance use clinic. The plan was for the patient to wear an Aspen cervical collar, complete a 14-day course of sulfamethoxazole-trimethoprim, and follow up as an outpatient in the Spine/Orthopedic surgery clinic.

## Discussion

C-spine fractures are of high concern in trauma because they may result in significant morbidity and mortality. The cervical spine is important in that it provides structural skeletal support, mobility of the neck region, and protection of nerves. While c-spine fractures may occur in all age groups, the injuries are more common in males. The most common causes of fractures include falls, motor vehicle accidents (MVC), biking, and diving.^3^

Upper cervical spine (C1–C2) trauma represents 25 to 40% of cervical injuries with a mortality of 8.4%.^4^ A C1, or atlas, fracture often results from axial loading. C2 fractures typically occur due to a mix of compression, hyperflexion, and hyperextension.^3^ Due to the C1–C2 joint’s high mobility, the nearby vertebral arteries are subject to dissection and occlusion, and therefore it is important to evaluate vasculature in patients with a history of trauma.^5^ Patients with atlas fractures usually present with pain in the upper cervical spine, with associated muscle tenderness, muscle spasms, and decreased range of motion of the upper cervical spine, especially with rotation.^6^ Neurological complications are rare with atlas fracture unless there is a retro-pulsed fragment compressing the spinal cord.^6^

When evaluating a potential c-spine injury, low-risk patients may be screened with the Canadian C-Spine Rule (Figure 1) which considers factors such as age, exam findings and mechanism of injury.^7^ The algorithm helps to avoid unnecessary radiation exposure to patients while also limiting the use of hospital resources.^8^

When upper cervical spine injury is suspected, computed tomography (CT) scans are critical to initial ED evaluation to look at the atlas ring structure and detect any bony avulsions.^3^ CT imaging is preferred to cervical spine plain radiography because CT has a sensitivity of 98% (95% CI 96,99%), vs 52% (95% CI 47,56%) for plain radiography for detecting fractures.^6^ Angio-CT or angio-MRI are recommended when vertebral artery injuries are suspected in cases involving transverse process lesions and lateral mass fracture that involve the foramen of the vertebral artery.^6^

After imaging and evaluation, patients are stratified based on the stability of their injury. If the fracture is unstable, hospital admission and consultation with the spine surgery team are needed. Patients with unstable fractures also commonly experience multisystem trauma which needs additional monitoring or intensive care based on the severity. For patients with a stable injury (i.e., minor spinal fracture patterns with no neurological deficits), outpatient management may be possible with warranted spine surgery consultation.

Our case shows a patient with cervical spine injury due to recreational wrestling, but C1 fractures alone do not have any unifying guidelines for treatment.^9^ The most important factor to consider is fracture stability. If the fracture is stable, nonoperative treatment is recommended with external orthoses such as rigid collars, halo-thoracic braces, or sternal occipital mandibular immobilizers. For patients with unstable fractures or who have neurologic sequalae, surgery may be considered, but for the majority of cases, a hard collar is considered adequate. For C2 fractures, non-odontoid fractures (hangman fracture), type I (fractures through the tip) and type III (fractures through the C2 vertebral body) odontoid fractures, treatment is nonsurgical via cervical orthoses. Type II (fracture through the base) odontoid fractures have variability in treatment based on practice. Recent study suggests that regardless of the type of fracture, age and degree of fracture displacement should be considered for determining surgery.^10^

Once the patient has been evaluated, efforts should be made to increase compliance with medical advice and treatment. This case demonstrates the importance of addressing polysubstance abuse with patients. Risk factors for patients leaving AMA after trauma include substance use and alcohol use disorder and patients are often readmitted with the need for additional care. Unique interventions in the above case included the involvement of pain medicine and social work during the hospital stay, treatment of dependence on multiple substances, and scheduled follow-up at a substance use clinic. Identifying patients who are at high risk for leaving AMA and creating an individualized approach to care may increase medical compliance, decrease hospital costs associated with readmissions, and improve patient care and prognosis.^11^

## Supplementary Information


















