# Supplementary figures and images for: A Case Report of an Unstable C-spine Fracture in the Emergency Department
Source: J Educ Teach Emerg Med. 2025 Apr 30;10(2):V1–5. doi: 10.21980/J8SK90 (PMC12054094; doi:10.21980/J8SK90)

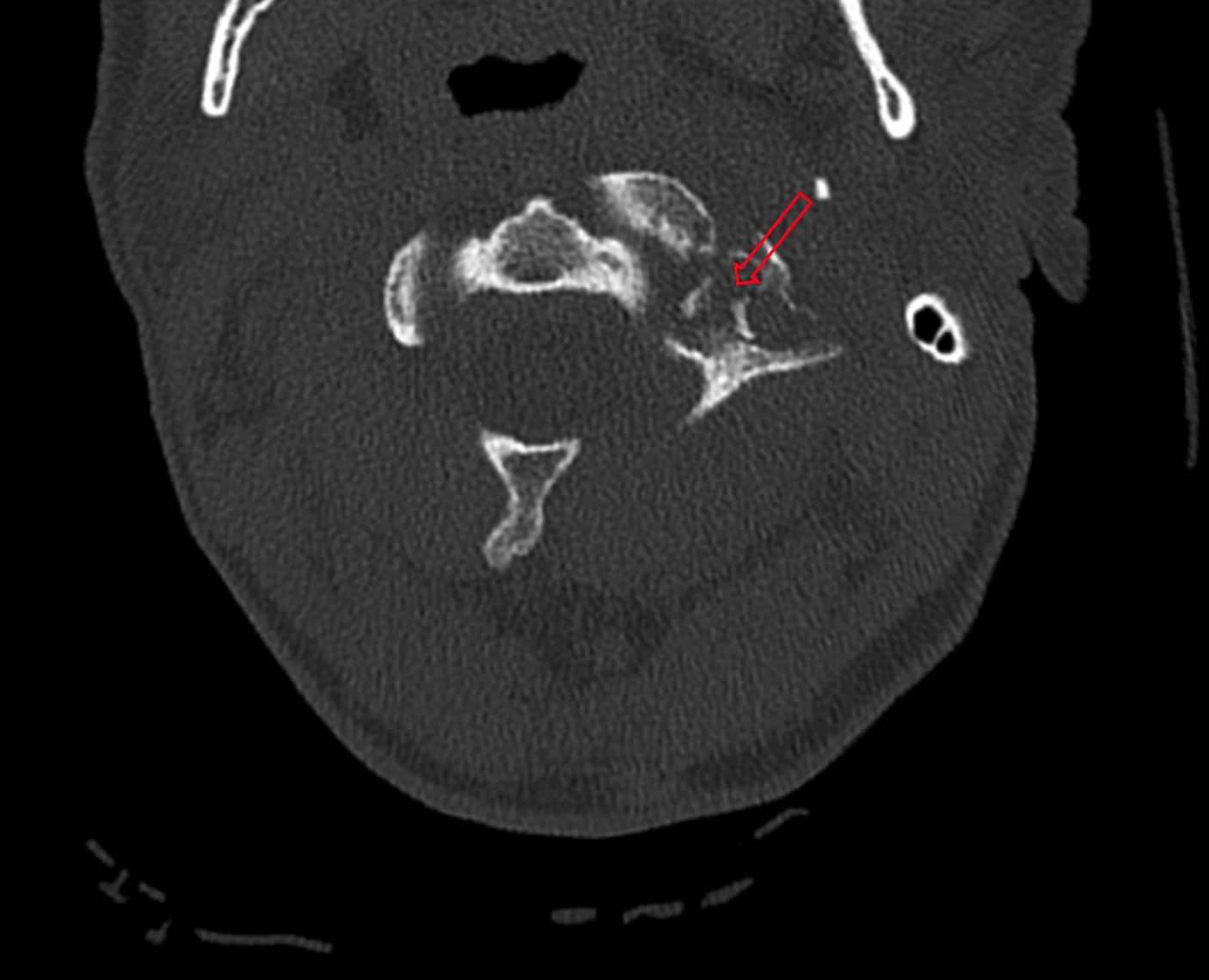

Supplement: Supplementary file 1 [file 10-2-V1-supp1.png]

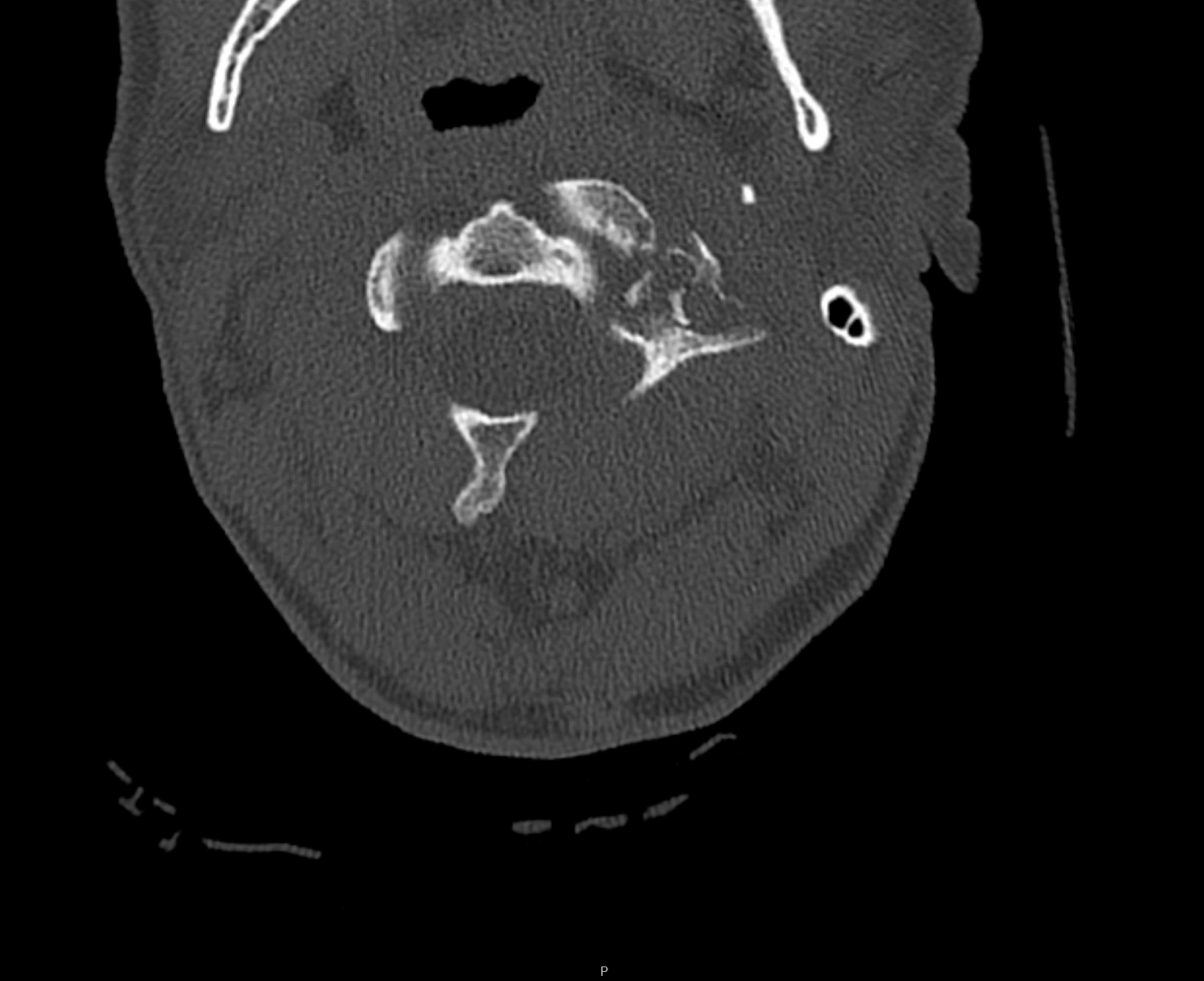

Supplement: Supplementary file 2 [file 10-2-V1-supp2.png]

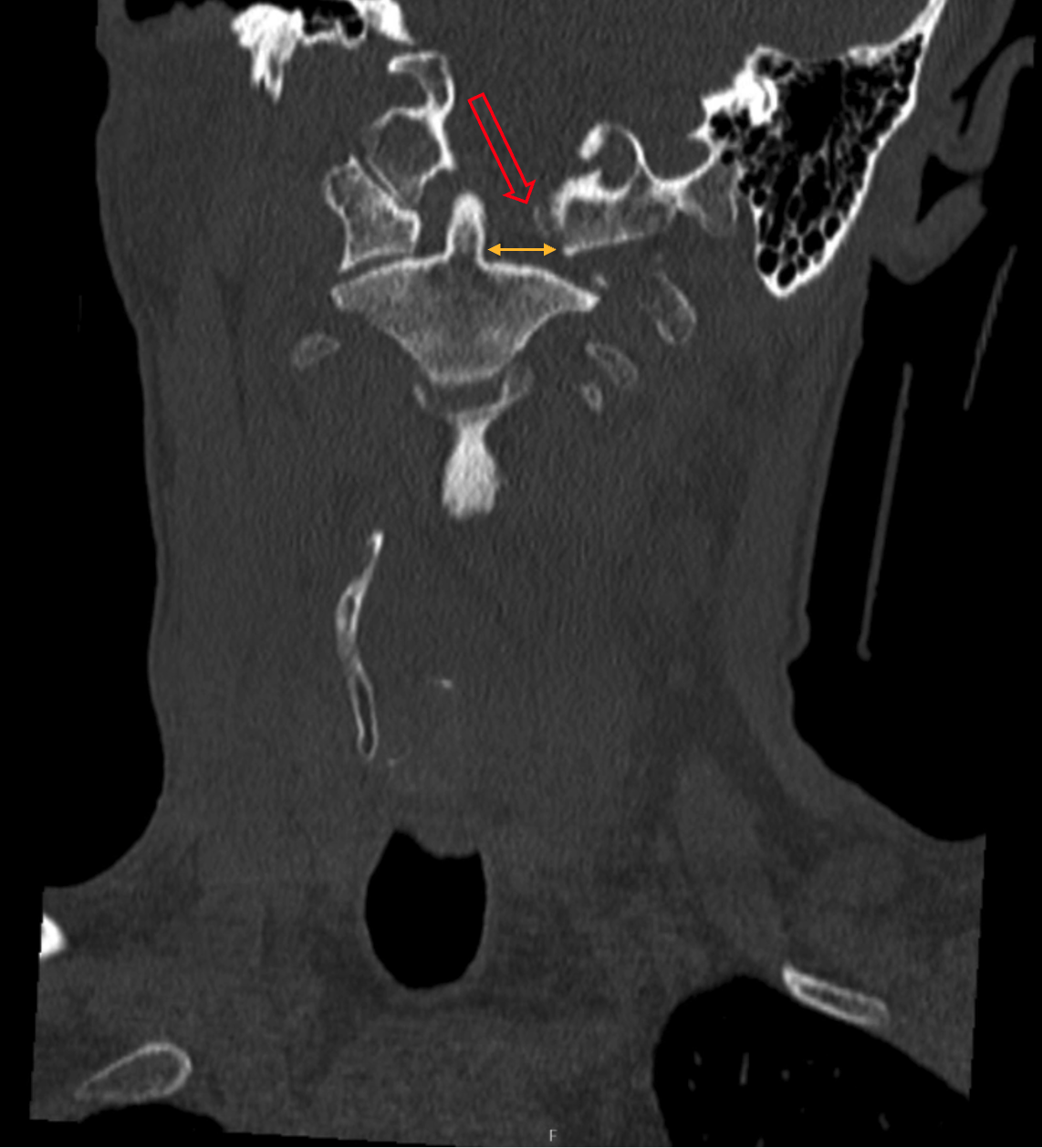

Supplement: Supplementary file 4 [file 10-2-V1-supp4.png]

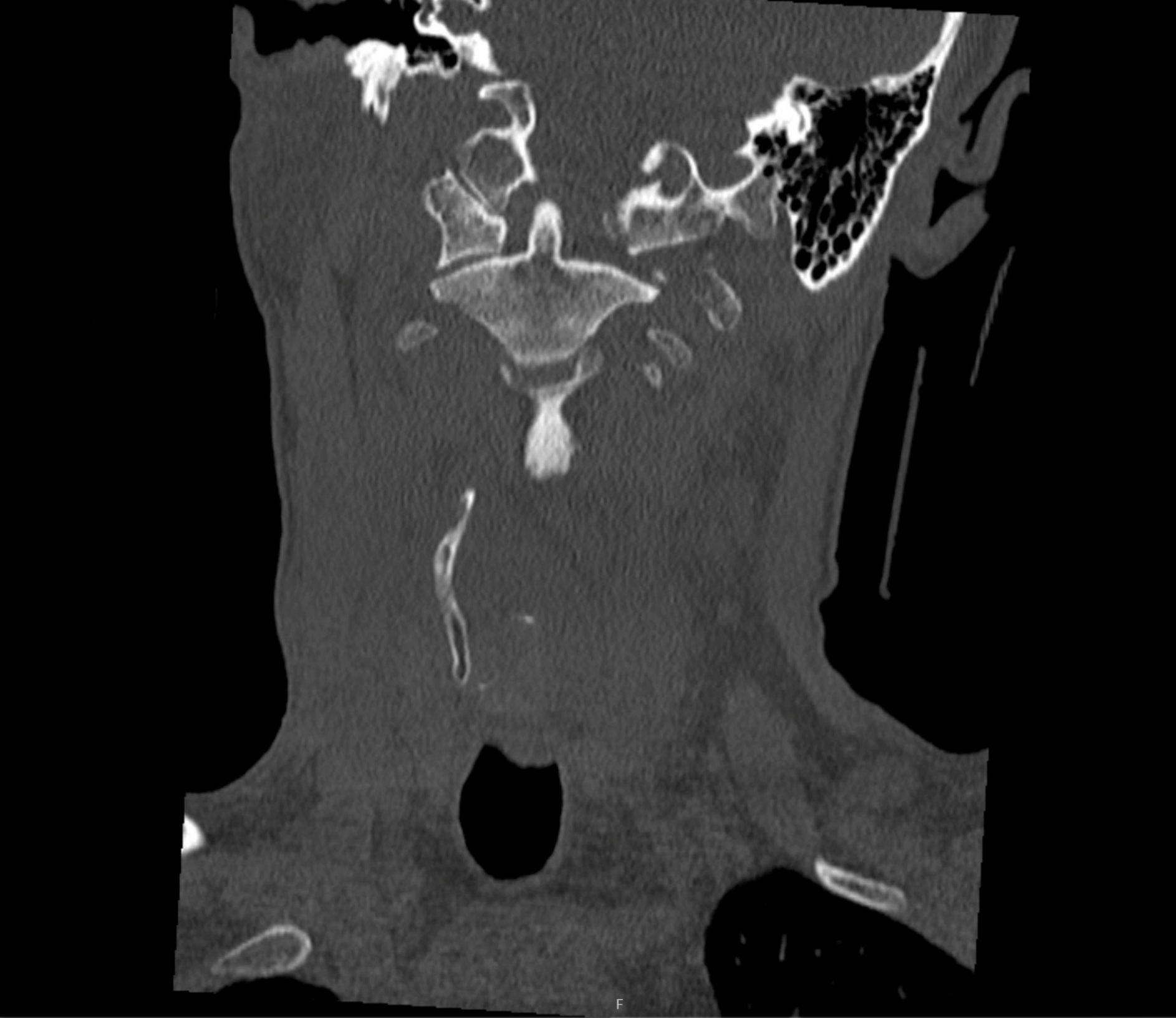

Supplement: Supplementary file 5 [file 10-2-V1-supp5.png]

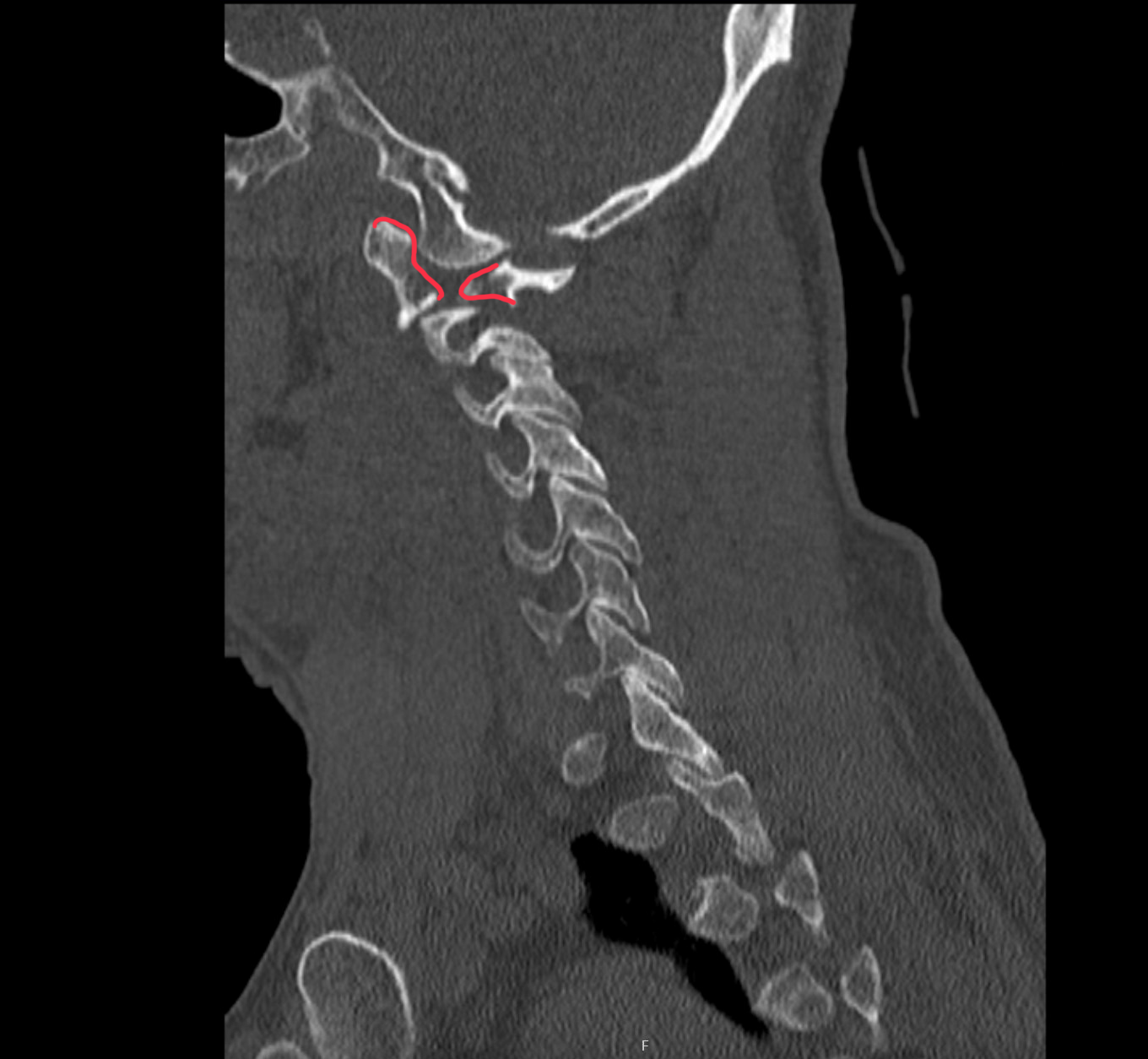

Supplement: Supplementary file 7 [file 10-2-V1-supp7.png]

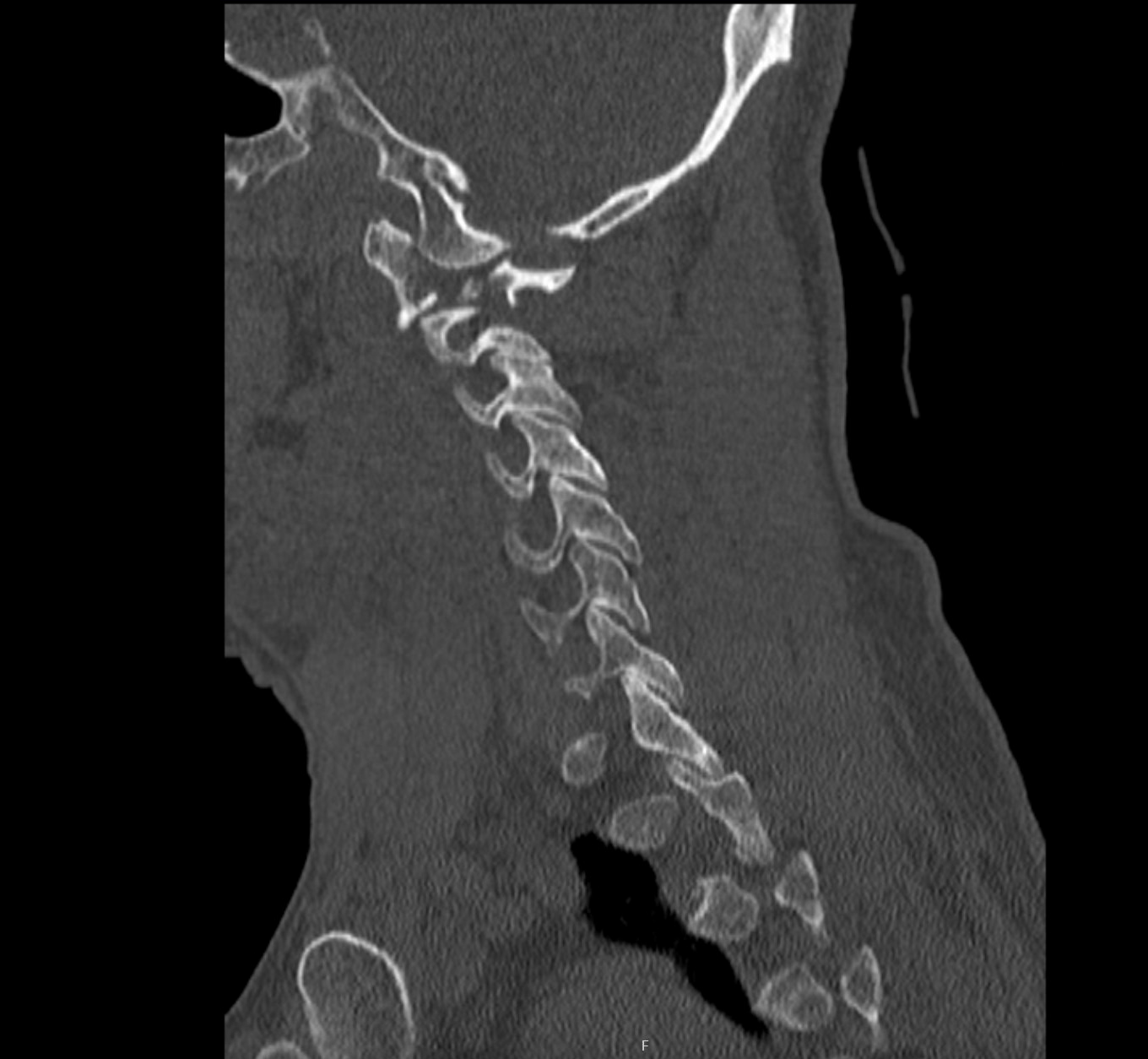

Supplement: Supplementary file 8 [file 10-2-V1-supp8.png]
